# A novel stratification framework based on anoikis-related genes for predicting the prognosis in patients with osteosarcoma

**DOI:** 10.3389/fimmu.2023.1199869

**Published:** 2023-07-27

**Authors:** Xiaoyan Zhang, Zhenxing Wen, Qi Wang, Lijuan Ren, Shengli Zhao

**Affiliations:** ^1^ Department of Spine Surgery, The First Affiliated Hospital of Sun Yat-Sen University, Guangzhou, China; ^2^ Department of Nutrition, College of Public Health of Sun Yat-Sen University, Guangzhou, China; ^3^ Guangdong Provincial Key Laboratory of Orthopaedics and Traumatology, Guangzhou, China; ^4^ Department of Oncology, Nanyang Central Hospital, Nanyang, China; ^5^ Molecular Diagnosis and Gene Testing Center, The First Affiliated Hospital of Sun Yat-Sen University, Guangzhou, China

**Keywords:** osteosarcoma, anoikis, prognosis, immune microenvironment, immunotherapy

## Abstract

**Background:**

Anoikis resistance is a prerequisite for the successful development of osteosarcoma (OS) metastases, whether the expression of anoikis-related genes (ARGs) correlates with OS prognosis remains unclear. This study aimed to investigate the feasibility of using ARGs as prognostic tools for the risk stratification of OS.

**Methods:**

The Cancer Genome Atlas (TCGA) and Gene Expression Omnibus (GEO) databases provided transcriptome information relevant to OS. The GeneCards database was used to identify ARGs. Differentially expressed ARGs (DEARGs) were identified by overlapping ARGs with common differentially expressed genes (DEGs) between OS and normal samples from the GSE16088, GSE19276, and GSE99671 datasets. Anoikis-related clusters of patients were obtained by consistent clustering, and gene set variation analysis (GSVA) of the different clusters was completed. Next, a risk model was created using Cox regression analyses. Risk scores and clinical features were assessed for independent prognostic values, and a nomogram model was constructed. Subsequently, a functional enrichment analysis of the high- and low-risk groups was performed. In addition, the immunological characteristics of OS samples were compared between the high- and low-risk groups, and their sensitivity to therapeutic agents was explored.

**Results:**

Seven DEARGs between OS and normal samples were obtained by intersecting 501 ARGs with 68 common DEGs. *BNIP3* and *CXCL12* were significantly differentially expressed between both clusters (*P*<0.05) and were identified as prognosis-related genes. The risk model showed that the risk score and tumor metastasis were independent prognostic factors of patients with OS. A nomogram combining risk score and tumor metastasis effectively predicted the prognosis. In addition, patients in the high-risk group had low immune scores and high tumor purity. The levels of immune cell infiltration, expression of human leukocyte antigen (HLA) genes, immune response gene sets, and immune checkpoints were lower in the high-risk group than those in the low-risk group. The low-risk group was sensitive to the immune checkpoint PD-1 inhibitor, and the high-risk group exhibited lower inhibitory concentration values by 50% for 24 drugs, including AG.014699, AMG.706, and AZD6482.

**Conclusion:**

The prognostic stratification framework of patients with OS based on ARGs, such as *BNIP3* and *CXCL12*, may lead to more efficient clinical management.

## 1 Introduction

Osteosarcoma (OS) is the most common primary bone malignancy that seriously threatens the health of children and adolescence worldwide (1). With the widespread use of multi-agent chemotherapy in the 1980s, the 5-year survival rate of patients with OS without metastasis has substantially increased from 20% to 65% (2, 3). However, the prognosis of patients with recurrence and metastasis remains dismal, with only a 12% 4-month event-free survival rate in single-arm phase II clinical trials (4). Owing to the heterogeneity of the tumor itself and the complex mechanism of metastasis, metastatic cells vary phenotypically from primary tumor cells and may more easily escape immune surveillance and survive chemotherapy (5, 6). This may explain why patients with the same clinical or pathological conditions, who receive the same treatment regimen, may have different clinical outcomes. Additionally, the lack of reliable markers and therapeutic targets makes it difficult to identify patients who are at a high risk of metastasis and may maximally benefit from specific therapies. Therefore, for a considerable period, the prediction and risk stratification of distant metastases before treatment remain the cornerstone for therapeutic decisions.

Anoikis is a type of programmed cell death caused by cell detachment from the extracellular matrix (ECM) (7). As a protective mechanism, anoikis can prevent the ectopic growth of somatic cells at inappropriate body sites. However, tumor cells with malignant potential usually show resistance to anoikis, allowing them to escape from the primary tumor site and colonize secondary sites (8). Specific factors, such as the integrin family, growth factor family, and metabolic intermediates have been reported as drivers of anoikis resistance, thus enhancing tumor recurrence and metastasis (7, 9). With the rapid development of omics technology and bioinformatic approaches, accumulating evidence suggests that tumor metastasis is tightly regulated at multiple levels by anoikis-related genes (ARGs). These genes remodel tumor cells by reprogramming lipid and amino acid metabolism to promote anoikis resistance. For example, the GDH1-mediated metabolic reprogramming of glutaminolysis mediates lung cancer metastasis (10). Upon TGF-β2 stimulation, PKC-zeta-mediated translocation of CD36 facilitates the uptake of fatty acids by tumor cells and supports their invasiveness (11). Advances in research on ARGs have provided new opportunities to clarify the mechanisms of tumor metastasis. However, to the best of our knowledge, few studies are available on the role of ARGs in OS metastasis. However, whether abnormal expression of ARGs is associated with poor OS prognosis has not been fully explored (5). The identification and characterization of ARGs in OS may provide new biomarkers and therapeutic targets for diseases.

Considering the potential correlations among ARGs, OS metastasis, and clinical outcomes, this study focused on the identification of ARGs that may influence the prognosis of OS. Following prognosis-associated clinical parameter screening, a prognostic risk model was established, and its ability to identify high-risk populations with OS was evaluated. Finally, we explored the specific immunological features of the high- and low-risk patients. The results of this study provide valuable references for clinicians to stratify at-risk patients with OS, which may lead to more efficient clinical management.

## 2 Materials and methods

### 2.1 Data source

A TARGET-OS dataset containing transcriptomic data of 85 OS samples with survival information was obtained from The Cancer Genome Atlas (TCGA) database (https://portal.gdc.cancer.gov/). The GSE16088, GSE99671, GSE19276, and GSE16091 datasets were acquired from the Gene Expression Omnibus (GEO) database (https://www.ncbi.nlm.nih.gov/geo/). The GSE16088 dataset contained 14 OS and 6 normal samples. The GSE99671 dataset contained 18 OS and 18 normal samples. The GSE19276 dataset contained 23 OS and 5 normal samples. The GSE16091 dataset contained 34 OS samples. In addition, 501 ARGs were identified and screened from the GeneCards database (https://www.genecards.org), and the screening conditions were a relevance score >0.4.

### 2.2 Identification of differentially expressed ARGs among OS and normal samples

Using the *limma* R package (Version 3.50.1), differentially expressed genes (DEGs) in samples from the OS and normal sample groups were obtained from the GSE16088 and GSE19276 datasets with the criteria of |logFC| > 1 and adj.P.Value < 0.05. In the GSE99671 dataset, DEGs among the OS and normal sample groups were screened using the DESeq2 R package (Version 1.34.0), and the identification thresholds were |logFC| > 1 and adj.P.Value < 0.05. The upregulated DEGs from the GSE16088, GSE19276, and GSE99671 datasets were crossed to obtain common upregulated DEGs. Similarly, common down-regulated DEGs in the above three datasets were obtained. Common upregulated and downregulated DEGs were combined to obtain DEGs between OS and normal samples. The combined DEGs were intersected with ARGs to obtain DEARGs.

### 2.3 Identification of anoikis-related clusters in patients with OS

Consistent clustering analysis was performed on samples from the TARGET-OS dataset based on DEARGs using the R package *ConsensusClusterPlus* (version 1.58.0) (12). The consistency clustering effect was assessed using principal component analysis (PCA). Next, the clinical characteristics of the different clusters of patients with OS were analyzed using the Chi-square test. In addition, differences in functional enrichment between different clusters were compared using the gene set variation analysis (GSVA) R (version 1.42.0) (13).

### 2.4 Risk model of patients with OS

Univariate Cox analysis of DEARGs in the TARGET-OS dataset was used to identify prognosis-related genes of patients with OS. Prognosis-related genes were tested for the proportional hazards (PH) hypothesis, and a multifactorial Cox model was constructed (14). Based on the median risk score, the patients with OS were classified into high- and low-risk groups. Kaplan-Meier (K-M) survival curves were used to compare the survival probability of patients with OS between the low- and high-expression groups based on the median expression value of each prognostic-related gene (15).

In the TARGET-OS dataset, differences in survival between patients in the two risk groups were compared using K-M survival curves. The receiver operating characteristic (ROC) curve of the risk score was plotted using the survivalROC R package (Version 3.2-13) (16). In addition, differences in the clinical traits of patients with OS among the risk groups were explored using the TARGET-OS dataset, and the association between the clinical characteristics and risk scores of patients with OS was assessed.

### 2.5 Nomogram model and GSVA between the high- and low-risk groups

In the TARGET-OS dataset, the independent prognostic value of clinical characteristics and risk scores was explored using univariate and multifactorial Cox analyses. Then, a nomogram model was obtained by combining factors with independent prognostic values, and the reliability of the nomogram model was assessed using decision curve analysis (DCA), ROC curves, and calibration curves (16). The GSVA was performed on samples from both risk groups.

### 2.6 Immune infiltration landscape

In the TARGET-OS dataset, the estimate R package (version 1.0.13) was used to compare stromal scores, immune scores, ESTIMATE scores, and tumor purity in samples from the high- and low-risk groups. The infiltration level of 30 tumor microenvironment (TME) cells in samples from both groups was assessed using single-sample gene set enrichment analysis (ssGSEA) (17). Correlations between differential immune cells and risk scores were calculated using Spearman analysis. In addition, the survival probability of patients with OS in the two groups of each TME cell type, according to the median ssGSEA score, was evaluated using K-M survival analysis. TME cells with different infiltrations between the two groups, TME cells that were related to the survivability of patients with OS, and TME cells related to the risk score were intersected to obtain important TME cells for the risk scores of patients with OS, as described above.

### 2.7 Immune microenvironment

First, immune-related genes were acquired from the ImmPort database (https://www.immport.org), and their expression in samples from the high- and low-risk groups was assessed using the TARGET-OS dataset. Correlations between prognostic- and immune-related genes were assessed using Pearson correlation analysis. Differences in the expression of human leukocyte antigen (HLA) genes between samples from both groups were compared, and correlations between prognostic-related genes and HLA genes were assessed. In addition, differences in the expression of immune checkpoints between both groups were evaluated, and the correlations between immune checkpoints and risk scores were further explored.

### 2.8 Sensitivity of patients with OS to therapeutic drugs

Treatment sensitivity to PD-1 and CTLA4 inhibitors was predicted in patients with OS from high- and low-risk groups using the SubMap algorithm in the TARGET-OS dataset (18). Differences in the 50% inhibitory concentration (IC50) of 138 drugs between both groups were compared using the pRRophetic algorithm (19).

## 3 Results

### 3.1 Screening for DEARGs among OS and normal samples

**Figure 1:**
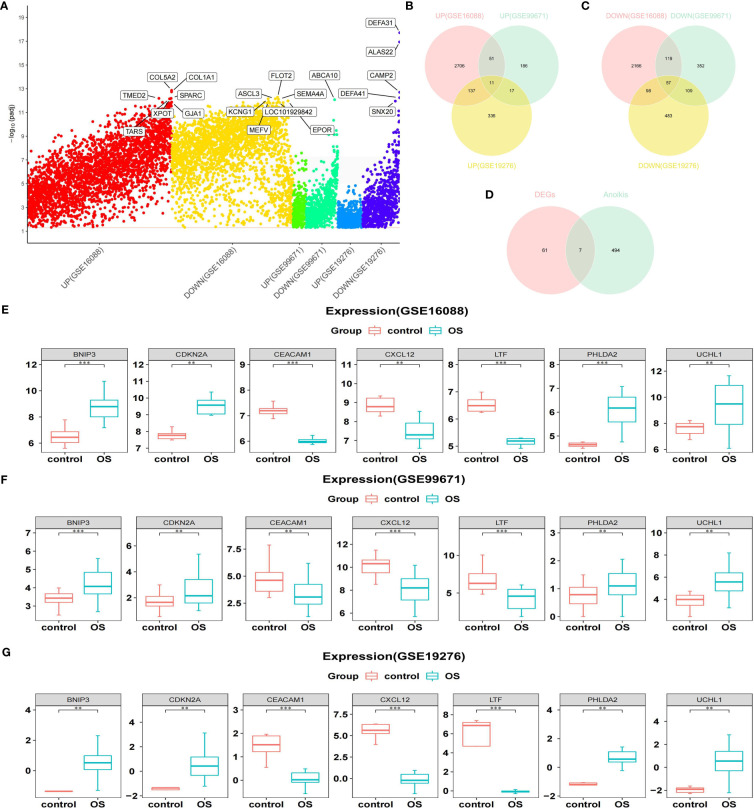
Acquisition of DEARGs. **(A)** Manhattan plot for DEGs in the GSE16088, GSE99671, and GSE19276 datasets. **(B, C)** Venn diagrams show the intersection of the number of URGs and DRGs among the above three databases. **(D)** Venn diagram shows the intersection of the number of DEARGs between DEGs and ARGs. **(E–G)** Boxplots show the consistent expression patterns of DEARGs among the above three databases. DEARGs, differentially expressed anoikis-related genes; DEGs, differentially expressed genes; URGs, up-regulated genes; DRGs, down-regulated genes; ARGs, anoikis-related genes.

Through differential analysis, 5345 DEGs among OS and normal samples were obtained from the GSE16088 dataset, comprising 2905 up-regulated genes (URGs) and 2440 down-regulated genes (DRGs). A total of 902 DEGs between OS and normal samples were obtained from the GSE99671 dataset, comprising 265 URGs and 637 DRGs. A total of 1248 DEGs between OS and normal samples were screened in the GSE19276 dataset, comprising 501 URGs and 747 DRGs (**Figure 1A**).

Genes screened from the three datasets with upregulated expression were crossed to acquire 11 commonly upregulated DEGs (**Figure 1B**). Similarly, genes that were downregulated in the three datasets were crossed to acquire 57 common downregulated DEGs (**Figure 1C**). Combining the common URGs and DRGs yielded 68 DEGs between the OS and normal samples.

Next, 68 DEGs between OS and normal samples were intersected with 501 ARGs to obtain seven DEARGs (including *BNIP3*, *CDKN2A*, *CEACAM1*, *CXCL12*, *LTF*, *PHLDA2*, and *UCHL1*) (**Figure 1D**). Among them, the expression of *BNIP3*, *CDKN2A*, *PHLDA2*, and *UCHL1* was significantly increased in OS samples, whereas the expression of *CEACAM1*, *CXCL12*, and *LTF* was significantly reduced compared to that normal samples (**Figures 1E–G**).

### 3.2 Acquisition of anoikis-related clusters of patients with OS

**Figure 2:**
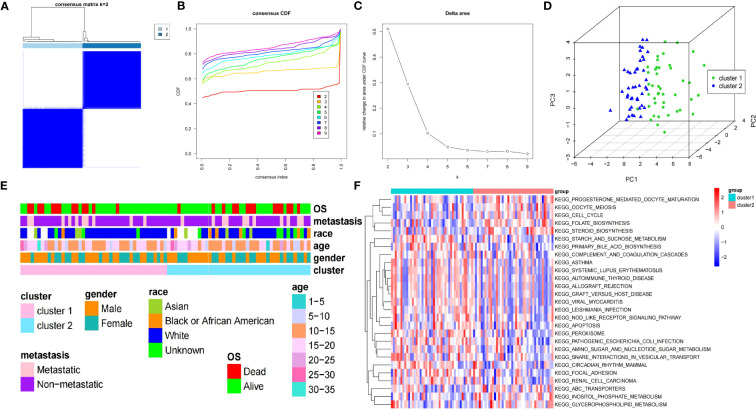
Clinical features and GSVA of two clusters of patients with OS. **(A)** Consensus matrix heatmap defines two OS clusters (k=2). **(B)** CDF curve for k = 2 – 9. **(C)** Relative change in the area under the CDF curve for k = 2 – 9. **(D)** PCA displays the different distribution of both clusters. **(E)** The clinical features between both clusters of patients with OS. **(F)** Heatmap of GSVA enrichment analysis results. GSVA, gene set variation analysis; OS, osteosarcoma; CDF, cumulative distribution function; PCA, principal component analysis.

According to the seven DEARGs, consistent cluster analysis of the TARGET-OS dataset samples showed that clustering was the best when K=2 and all samples were classified into cluster 1 and cluster 2 (**Figures 2A–C**). PCA showed good results for consistent clustering, with all samples clustered into two clusters (**Figure 2D**). The race of patients with OS in the different clusters was significantly different (*P*<0.05); however, no significant difference was detected in the other clinical characteristics between both clusters (**Figure 2E**). In addition, 27 pathways were enriched between both clusters by GSVA, including progesterone-mediated oocyte maturation, oocyte meiosis, cell cycle, folate biosynthesis, and steroid biosynthesis (**Figure 2F**).

### 3.3 Prognosis-related gene expression levels were significantly different in OS and normal samples

**Figure 3:**
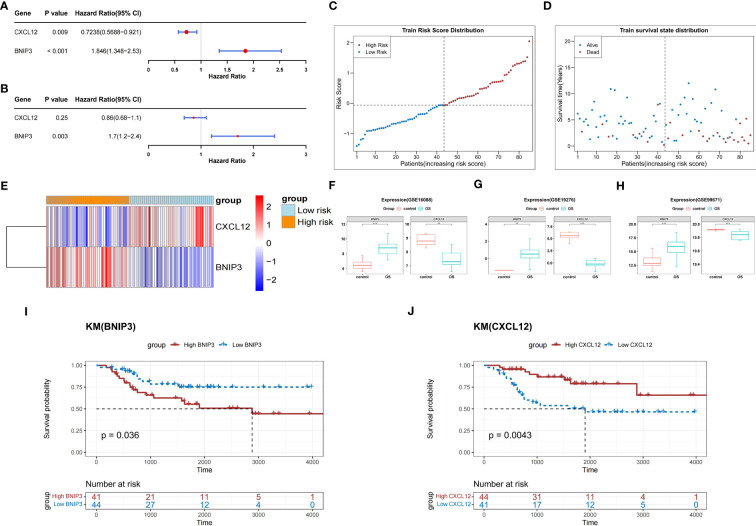
Construction of a risk score model based on two prognosis-related genes. **(A)** Forest plot of univariate Cox analysis. **(B)** Forest plot of multifactorial Cox analysis. **(C)** The risk score distribution of the training set. **(D)** Distribution of survival status in patients with OS. **(E)** The relative expression level of two prognosis-related genes in two risk groups. **(F–H)** Boxplots show the consistent expression patterns of two prognosis-related genes among the above three databases. **(I)** K-M survival analysis shows the relationship between *BNIP3* expression and OS prognosis. **(J)** K-M survival analysis shows the relationship between *CXCL12* expression and OS prognosis. OS, osteosarcoma; K-M, Kaplan-Meier.

In the TARGET-OS dataset, two prognosis-related genes (*CXCL12* and *BNIP3*) were identified by univariate Cox analysis of the seven DEARGs (*P*<0.05) (**Figure 3A**). Both *CXCL12* and *BNIP3* satisfied the PH hypothesis (*P*<0.05) and were used to construct a multifactorial Cox model (**Figure 3B**). Patients in the high-risk group with relatively short survival times accounted for most patients with OS (**Figures 3C, D**). *CXCL12* was strongly expressed in the low-risk group, whereas *BNIP3* was substantially expressed in the other group (**Figure 3E**). In the GSE16088, GSE19276, and GSE99671 datasets, the expression level of *BNIP3* was higher and that of *CXCL12* was lower in the OS samples than those in the normal samples (adj.*P* < 0.05) (**Figures 3F–H**).

K-M survival curves demonstrated that, compared with that of the other groups, the probability of survival in patients from the *BNIP3* high-expression group in the TARGET-OS dataset was greatly reduced (*P*<0.05) (**Figure 3I**). In contrast, the probability of survival in patients from the *CXCL12* high expression group was significantly higher (*P*<0.05) (**Figure 3J**).

### 3.4 Survival probabilities for patients in the high-risk group were substantially lower compared to those in the low-risk group

**Figure 4:**
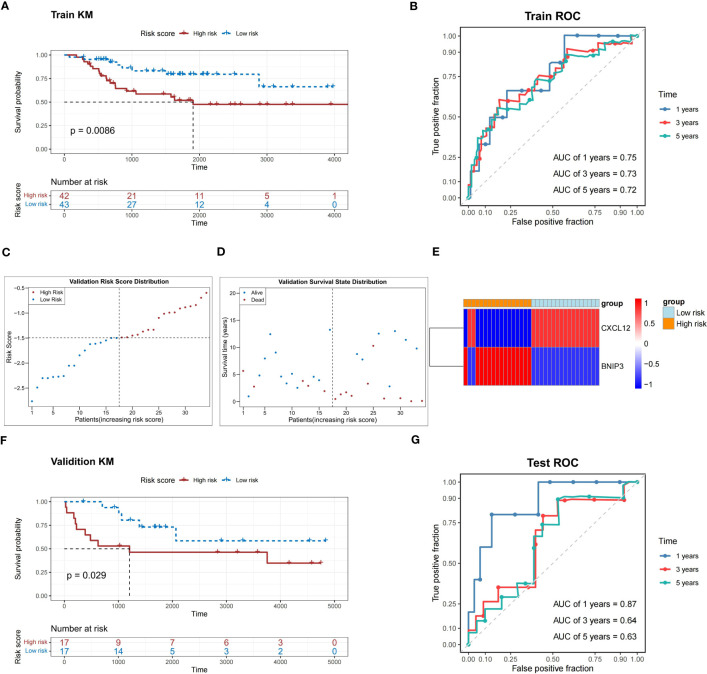
Performance assessment of the prediction model in the training and validation sets. **(A)** K-M survival analysis shows a significant difference in survival between the high- and low-risk groups in the training set. **(B)** ROC curves show the prediction power of the risk score in the training set for 1, 3, and 5 years. **(C)** The risk score distribution of the validation set. **(D)** Distribution of the survival status in patients with OS. **(E)** The relative expression level of two prognosis-related genes in two risk groups. **(F)** K-M survival analysis shows a significant survival difference between the high- and low-risk groups in the validation set. **(G)** ROC curves show the prediction power of the risk score in the validation set for 1, 3, and 5 years. K-M, Kaplan-Meier; ROC, receiver operator characteristic; OS, osteosarcoma; AUC, area under the curve.

In the TARGET-OS dataset, the probability of survival was greatly reduced for patients in the high-risk group compared to that in the other groups (*P*<0.05) (**Figure 4A**). The area under the ROC curve (AUC) of the risk model was 0.7, indicating that the risk model had a strong predictive ability for TARGET-OS dataset patients (**Figure 4B**).

In the validation dataset (GSE16091), high-risk patients with relatively short survival times accounted for most patients with OS (**Figures 4C, D**). The expression patterns of *CXCL12* and *BNIP3* in the high- and low-risk groups were similar to those observed in the TARGET-OS dataset (**Figure 4E**). The probability of survival was greatly reduced in patients in the high-risk group compared to those in the other groups (*P*<0.05) (**Figure 4F**). Meanwhile, the risk model correctly estimated the prognosis of patients with OS because the ROC curves of the risk model in the GSE16091 dataset were all greater than 0.6 (**Figure 4G**).

### 3.5 OS patients of Black or African American and metastatic OS patients had a high-risk score

**Figure 5:**
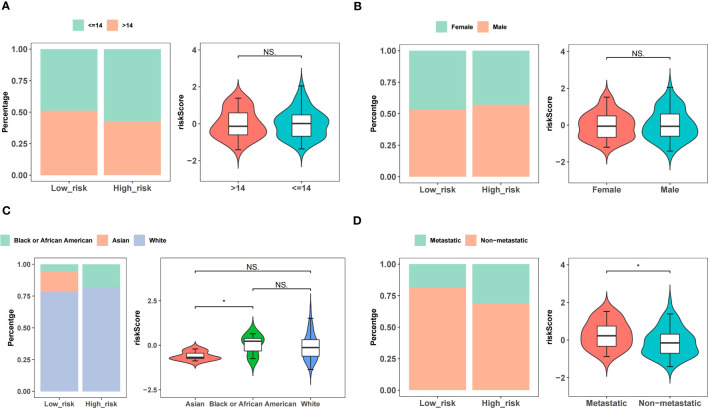
Correlations between risk scores and clinical features. **(A)** Age. **(B)** Gender. **(C)** Race. **(D)** Metastatic or non-metastatic.

In the TARGET-OS dataset, the race of the patients with OS differed between the high- and low-risk groups, whereas other clinical characteristics did not significantly differ (**Table 1**). Age and sex did not strongly correlate with the risk scores of patients with OS (**Figures 5A, B**). Black or African Americans had significantly higher risk scores than those Asian patients (**Figure 5C**). Patients with metastatic OS had substantially higher risk scores than those with nonmetastatic OS (**Figure 5D**).

**Table 1:** Clinical features in the two risk groups.

	Total	Low-risk	High-risk	*P*-value
**Age**	85	43	42	
<=14	45	21	24	0.583
>14	40	22	18	
**Gender**	85	43	42	
Femal	38	20	18	0.904
Male	47	23	24	
**Race**	65	37	28	
Black or African American	7	2	5	0.032
Asian	6	6	0	
White	52	29	23	
**Metastatic**	85	43	42	
Metastatic	21	8	13	0.286
Non-Metastatic	64	35	29	

### 3.6 Nomogram model could reliably forecast the prognosis of patients with OS

**Figure 6:**
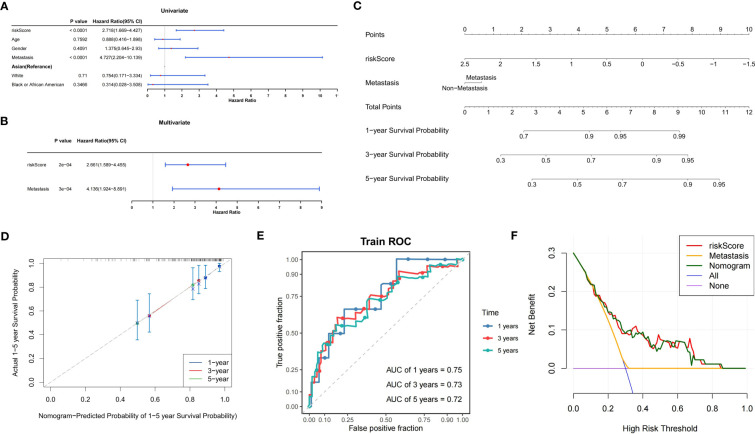
Construction of the prognostic model for OS. **(A)** Forest plot of univariate Cox analysis. **(B)** Forest plot of multifactorial Cox analysis. **(C)** Nomogram model for the prediction of survival possibility at 1, 3, and 5 years. **(D)** The calibration curve reveals the nomogram model has a good predictive ability. **(E)** Time-dependent ROC curves of overall survival at 1, 3, and 5 years. **(F)** DCA curves illustrating the clinical effectiveness of the nomogram model (purple line indicates patients were all alive; blue line indicates patients were all dead). OS, osteosarcoma; ROC, receiver operator characteristic; AUC, area under the curve; DCA, decision curve analysis.

Univariate Cox analysis confirmed the association between the risk score and metastasis and OS in the TARGET-OS dataset (*P*<0.05) (**Figure 6A**). Next, the independent prognostic value of the risk score and metastasis was identified using multifactorial Cox analysis (*P*<0.05) (**Figure 6B**).

To create a nomogram model, it was possible to reliably estimate OS prognosis by fusing the risk score with metastasis (**Figures 6C–E**). In addition, the DCA curves showed that the nomogram model had greater reliability in predicting the survival of patients with OS (**Figure 6F**).

### 3.7 The differentially expressed signaling pathways among high- and low-risk groups were mainly associated with tumorigenesis and metastasis

**Figure 7:**
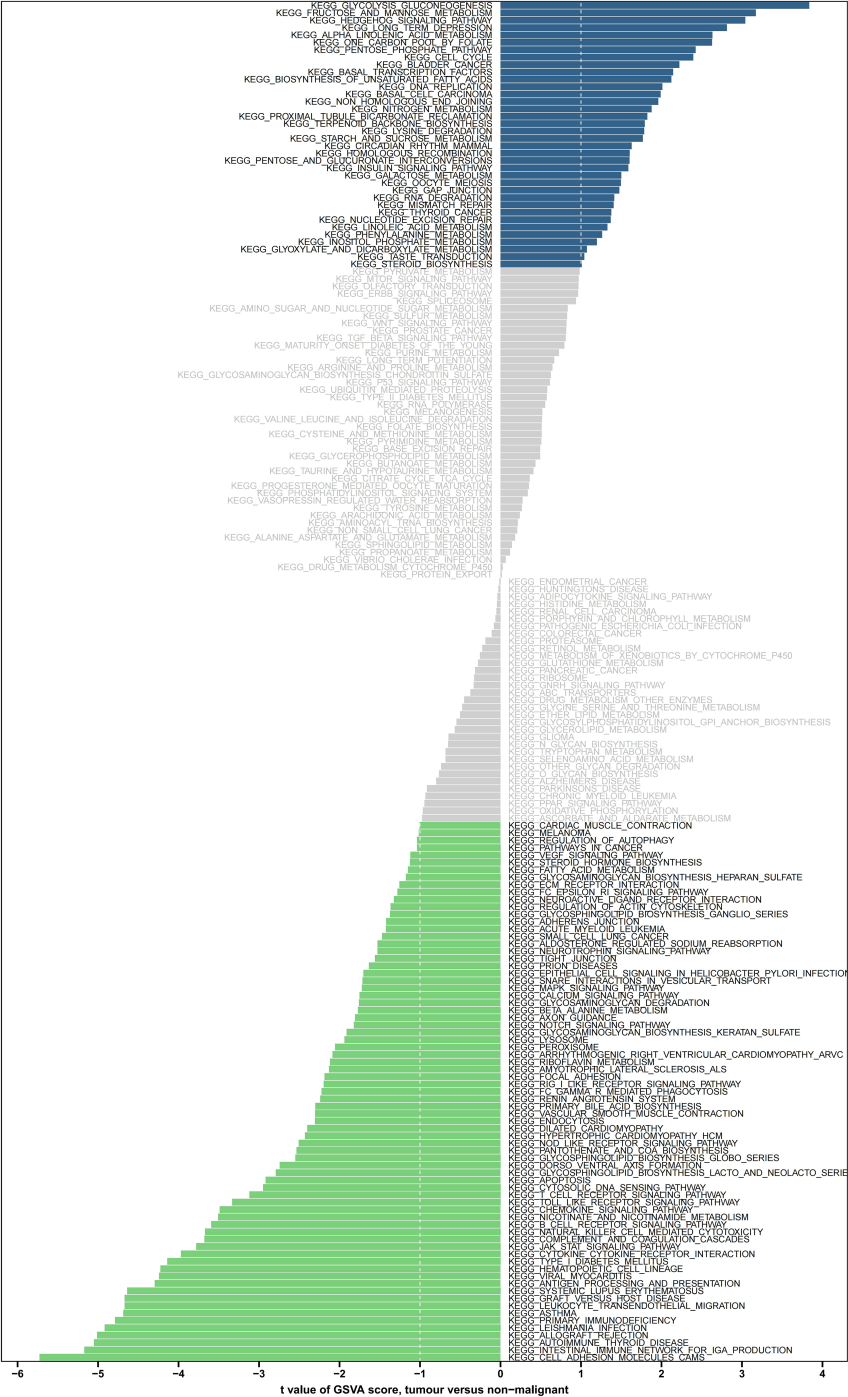
GSVA enrichment analysis of the biological pathways between the two risk groups. Green indicates significant down-regulation, blue indicates significant up-regulation, and gray indicates no significance. GSVA, gene set variation analysis.

The GSVA enriched 31 pathways that significantly differed between both risk groups, including IGA production facilitated by the intestinal immune network, thyroid disease of autoimmune origin, rejection of allografts, leishmaniasis, and cellular adhesion molecules (**Figure 7**).

### 3.8 High-risk score was characterized by low immune infiltration and elevated tumor purity

**Figure 8:**
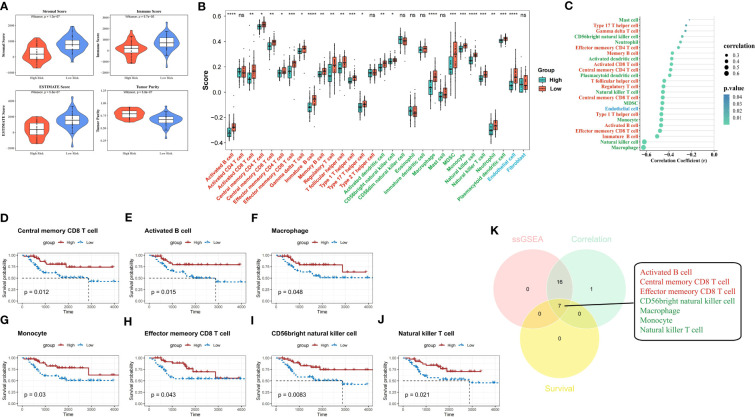
ESTIMATE analysis and hub TME cell screening. **(A)** Stromal score, immune score, ESTIMATE score, and tumor purity in different risk groups were evaluated by ESTIMATE analysis. **(B)** Immune infiltration levels of 28 immune cell types in the high- and low-risk groups (red indicates adaptive immune cell, green indicates innate immune cell, and blue indicates stromal cell). **(C)** The correlation between the risk score and 30 TME infiltration cells (red indicates adaptive immune cell, green indicates innate immune cell, and blue indicates stromal cell). **(D–J)** K-M survival analyses show the survivability probabilities in the high- and low-score groups of 30 TME cells (only statistically significant TME cells are visualized). **(K)** Venn diagram shows the hub TME cells (red indicates adaptive immune cell and green indicates innate immune cell). TME, tumor microenvironment; K-M, Kaplan-Meier.

In the TARGET-OS dataset, we detected that samples in the high-risk group had significantly lower stromal, immune, and ESTIMATE scores and significantly higher tumor purity than those of the samples in the other groups (*P*<0.05) (**Figure 8A**). The level of infiltration of 23 TME cells was significantly different from that of the other groups (*P*<0.05) (**Figure 8B**), and 24 TME cells were significantly correlated with risk scores (*P*<0.05) (**Figure 8C**). Individuals in the low-scoring groups of central memory CD8 T cells, activated B cells, macrophages, monocytes, effector memory CD8 T cells, CD56 bright natural killer cells, and natural killer T cells exhibited dramatically lower survival probabilities than those in the other groups (*P*<0.05) (**Figures 8D–J**).

The 23 TME cells that were differentially infiltrated between the two risk groups, the 24 TME cells that were related to the risk score, and the seven cells that were related to survival in patients with OS were intersected to obtain seven TME cells that were significant in the OS risk score (**Figure 8K**).

### 3.9 The expression of the immune response gene set and HLA genes immune checkpoints were significantly lower in the high-risk group of patients with OS

**Figure 9:**
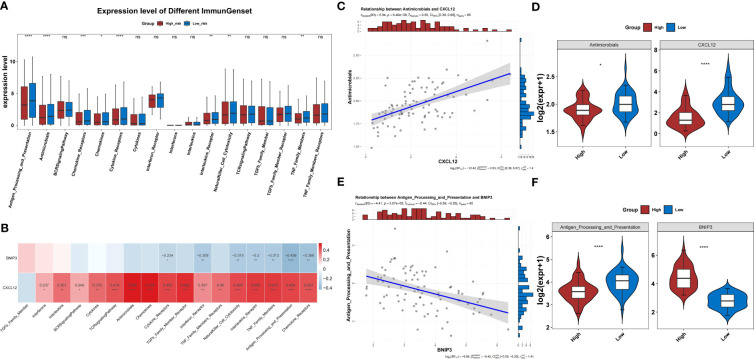
Immune response gene sets in the two risk groups. **(A)** Difference in the immune response between the high- and low-risk groups. **(B)** Correlation heatmap of two prognosis-related genes and immune response gene sets. **(C)** The level of *CXCL2* shows the most significant positive correlation with the antimicrobials gene set. **(D)** Differences in expression of the antimicrobials gene set between high- and low-risk groups. **(E)** The level of *BNIP3* shows the most significant negative correlation with the antigen processing and presentation gene set. **(F)** Differences in expression of the antigen processing and presentation gene set between high- and low-risk groups.

Eight immune response gene sets differed between both risk groups in the TARGET-OS dataset (**Figure 9A**). Immune response gene sets were negatively correlated with *BNIP3* expression and positively correlated with *CXCL12* expression (**Figure 9B**). The immune response gene sets most associated with *CXCL12* and *BNIP3* were both highly expressed in the low-risk group compared to those in the other groups (**Figures 9C–F**).

**Figure 10:**
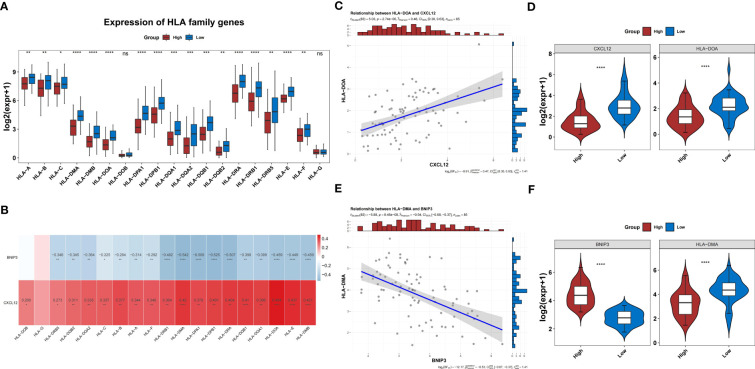
HLA families in the two risk groups. **(A)** Difference in the expression of HLA families between groups. **(B)** Correlation heatmap of two prognosis-related genes and HLA families. **(C)** The level of *CXCL2* shows the most significant positive correlation with HLA-DOA. **(D)** Differences in expression of HLA-DOA between high- and low-risk groups. **(E)** The level of *BNIP3* shows the most significant negative correlation with HLA-DMA. **(F)** Differences in expression of HLA-DMA between high- and low-risk groups. HLA, human leukocyte antigen.

**Figure 11:**
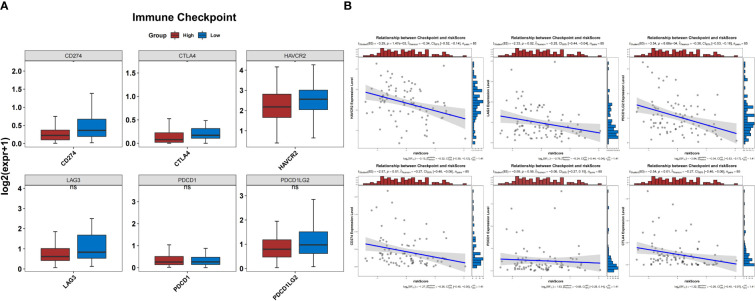
Prediction of response to immunotherapy. **(A)** The expression of three immune checkpoints was significantly different between the groups. **(B)** Correlation between the risk score and expression of immune checkpoints.

In the TARGET-OS dataset, the expression of 17 HLAs was highly variable between both groups (*P*<0.05) (**Figure 10A**). The HLAs were negatively associated with *BNIP3* and actively associated with *CXCL12* (**Figure 10B**). The HLAs were most strongly associated with *CXCL12* and *BNIP3*, and both were highly expressed in the low-risk group compared to those in the other groups (**Figures 10C–F**). In addition, the expression of *CD274*, *CTLA4*, and *HAVCR2* was noticeably lower in the high-risk group than that in the other groups (*P*<0.05) (**Figure 11A**). The risk score was negatively correlated with *HAVCR2*, *LAG3*, *PDCD1LG2*, *CD274*, and *CTLA4* (**Figure 11B**).

### 3.10 Patients with OS in the high-risk group had reduced sensitivity to therapeutic agents

**Figure 12:**
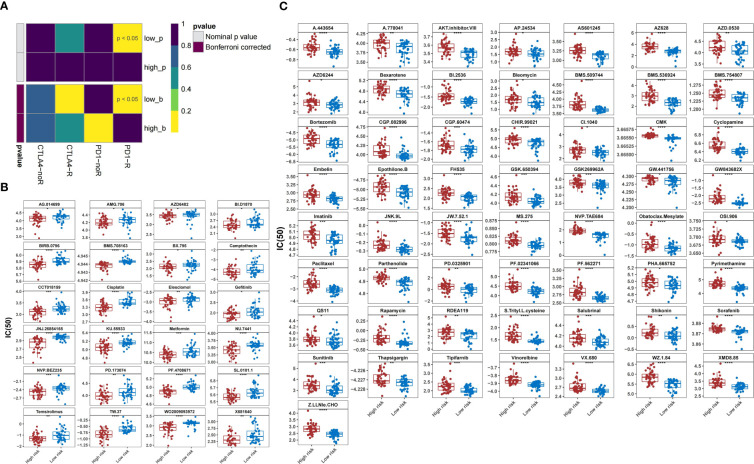
Prediction of response to therapeutic drugs. **(A)** Sensitivity of different groups to PD-1 and CTLA4 inhibitors. **(B)** Agents with lower IC50 in the high-risk group than those in the low-risk group. **(C)** Agents with higher IC50 in the high-risk group than those in the low-risk group. IC50, 50% inhibitory concentration.

Patients in the low-risk group were susceptible to PD-1 inhibitor treatment (*P*<0.05) (**Figure 12A**). A total of 81 drugs showed significantly different IC50 values between the two groups (*P*<0.05). Of these, 24 drugs, including AG.014699, AMG.706, AZD6482, and BI.D1870, may be candidates for treating patients in the high-risk group (**Figure 12B**). In contrast, 57 drugs, including A.443654, A.770041, AKT inhibitor VIII, and AP.24534, may not be ideal for patients in the other group (**Figure 12C**).

## 4 Discussion

Although the prognosis of localized OS has markedly improved owing to new therapeutic developments, long-term survival has stagnated over the past several decades (2–4). Metastasis, particularly lung colonization, is the most common cause of death in high-risk patients with OS (20). Anoikis is a physiological process that plays an important role in tissue homeostasis and development. Under pathological conditions, it is the main factor in tumor metastasis and therapy failure (7, 8). Alterations in ARGs that lead to anoikis resistance are hallmarks of the malignant transformation of tumors (10, 11, 21, 22). Regrettably, the relationship between ARGs and OS progression is much less recognized, which has limited the improvement in patient prognosis. The present study identified prognosis-related genes involved in OS progression. Based on *BNIP3* and *CXCL12* expression, this stratification framework can be used to effectively stratify the survival of patients with OS. Moreover, patients with high-risk OS presented unique patterns of immune characteristics and sensitivity to different chemotherapeutic agents. These findings provide a scientific basis for the discovery of new immunotherapeutic targets and efficient selection of existing drugs in clinical practice.

In this study, *CEACAM1*, *CXCL12*, and *LTF* were found to be downregulated in OS samples. CEACAM1 is a transmembrane cell adhesion molecule belonging to the CEA superfamily (23). Similar to the inhibitory signaling mode of PD-1, CEACAM1 represses the anti-tumor activity of T cells by dephosphorylating the downstream kinases of T cell receptor signaling (24–26). Monoclonal antibodies targeting CEACAM1 have been approved for the treatment of advanced and recurrent cancers in clinical trials (27). Downregulation of CXCL12 in OS facilitates the release of tumor cells from the bone and metastasis to other tissues (28). However, the protective coating formed by CXCL12 allowed malignant cells to escape immune attacks by T cells (29). After specific binding to CXCR4, the MAPK, PI3K, and phospholipase C pathways are activated, and the antitumor immune response is suppressed (30). LTF is widely considered a tumor suppressor. Consistent with the results of this study, patients with OS with low LTF levels showed lower survival rates, which may be attributed to the inhibitory effect of LTF on tumor cell proliferation (31).

*BNIP3*, *CDKN2A*, *PHLDA2*, and *UCHL1* are genes that we found highly expressed in OS samples. Upregulation of *BNIP3* has been reported to enhance anoikis resistance in hepatocarcinoma cells (32). A strong correlation between high BNIP3 levels and lower progression-free survival has also been observed in some platinum-resistant tumors (33). *CDKN2A* encodes a protein called p14ARF, which binds MDM2 in the nucleus and binds it to the nucleolus, thereby attenuating the ubiquitination degradation of p53 caused by MDM2. At the same time, it can accelerate the degradation of MDM2, increase the synthesis of p53 protein, and finally make tumor cells stagnated in G1/S phase, and play the tumor inhibition function (34, 35). Xie et al. observed a high-frequency *CDKN2A* mutation in the genomic profile of patients (36). The GA and AA genotypes of rs3217992 in *CDKN2A* may indicate higher malignancy, higher risk of lung metastasis, and poorer prognosis (37). *PHLDA2* encodes a pleckstrin homology domain-containing protein that inhibits cell proliferation by suppressing AKT activation (38). Decreased *PHLDA2* expression increases cell proliferation and reduces sensitivity to targeted agents in EGFR/ErbB2-driven cancer (39). How changes in *PHLDA2* affect the development of OS requires further investigation. UCHL1 is a de-ubiquitinating enzyme that has been found to be over-expressed in some cancers and is considered a cancer promoter. *UCHL1* downregulation decreases the proliferation, migration, and invasion of lung adenocarcinoma cells (40). It has also been proven to induce metastasis of breast cancer cells by acting on TGF-β signaling (41). The high expression of *UCHL1* in OS observed in this study suggests that it may play a role in tumor progression.

Changes in the relevant pathways in the differential genes of the low- and high-risk groups were further obtained by GSVA analysis, which showed that pathways, such as JAK-STAT, toll-like receptor, and Hedgehog signaling were substantially enriched. *STAT5* downregulation inhibits the proliferation, clonogenicity, and growth of OS cells (42). The toll-like receptor signaling pathway is remarkably differentially expressed in OS and is involved in the regulation of apoptosis, inflammation, and immunity (43). Similar to other studies, this study also confirmed the abnormality of the Hedgehog signaling pathway in OS, which may be related to tumor metastasis (44, 45). Further investigation of the regulatory mechanisms of signaling pathways in different risk groups may provide additional information to better understand the heterogeneity within tumors.

The TME broadly consists of tumor, immune, and stromal elements and has been proven to determine the biological behavior of tumor cells. As expected, we found a pattern of low scores of immune, stromal, and ESTIMATE, and a high score of tumor purity in high-risk groups, which is commonly observed in malignant solid tumors; this was confirmed by the significantly lower survival rate we found in high-risk populations (46–48). To further characterize immune infiltration between the groups, we identified seven TME cells that were substantially different in the OS samples. The number of these cells was low in the high-risk group and negatively correlated with the risk score. *BNIP3* overexpression accelerates the death of macrophages and T cells and promotes tumor proliferation and early metastasis (49, 50). CXCL12 is an important chemokine in T and NK cells that helps macrophages polarize into tumor-associated macrophages (51, 52). In addition, CXCL12 mediates the progression of rectal cancer by promoting the retention of neutrophils in tumors and increasing their interactions with CD8+ T cells (53). These findings not only suggest that low-level infiltration of immune cells in OS high-risk samples may be associated with poor prognosis but also highlight the non-negligible role of *BNIP3* and *CXCL12* in the regulation of immune cell biological behavior.

Besides immune cell infiltration, the immune response gene set and HLA family genes were also found to be differentially expressed between the two risk groups in our study. Through the correlation analysis, we found that antigen processing and presentation and HLA-DMA were the two entries that most negatively correlated with *BNIP3*, whereas antimicrobials and HLA-DOA were most positively correlated with *CXCL12*. HLA families participate in tumor immunity (54). The downregulation of HLA genes may reduce antigen presentation and facilitate immune evasion (55). HLA-DMA variants have been reported to be associated with a higher risk of local recurrence in head and neck squamous cell carcinoma (56). HLA-DOA is also a key molecule in the antigen processing and presentation pathway and has been implicated in OS progression through the downregulation of HLA-DOA expression (57, 58). Another study has described the inhibitory role of HLA-DOA in B cell-mediated antigen presentation (59). Based on the present evidence, we hypothesized that the immune dysregulation associated with *BNIP3* and *CXCL12* may drive the poor prognosis of OS.

In the last few decades, immune checkpoint inhibitor-based immunotherapies have provided a huge boost to research on immune surveillance and have transformed the therapeutic landscape of cancer (60). However, immune checkpoint blocking therapy is less effective in treating OS, with only 5% of patients with OS achieving objective remission in a 2017 clinical trial of the PD-1 antibody (61, 62). To clarify the differences in the response to immunotherapy between the high- and low-risk groups, we examined the expression levels of common immune checkpoints. We found that *CD274*, *CTLA4*, and *HAVCR2* were substantially under-expressed in the high-risk groups and were negatively correlated with risk scores. Similar to previous studies, patients in the high-risk group showed insensitivity to PD-1 and CTLA4 inhibitors, which may partially explain the poor response of some patients with OS to immunotherapy in clinical practice (62, 63). In addition, the 24 agents in our study showed sensitivity in high-risk groups and may help improve OS outcomes. In the future, combinations of immune checkpoint inhibitors with chemotherapy, targeted therapies, or novel therapies could potentially lead to new treatment strategies (2, 64, 65).

## 5 Conclusion

To the best of our knowledge, this is the first study to identify OS-related DEARGs and explore their predictive power for disease prognosis. The stratification framework based on *BNIP3* and *CXCL12* can effectively screen individuals at high risk for OS. Compared with those of the low-risk group, the high-risk group had unique immune characteristics and sensitivity to drug therapy, which may provide a scientific reference for clinicians to develop efficient treatment strategies. Further experimental and clinical studies based on our results are promising to consistently improve the prognosis of patients with high-risk OS.

## Data availability statement

The datasets presented in this study can be found in online repositories. The names of the repository/repositories and accession number(s) can be found within the article/supplementary material.

## Author contributions

SZ and LR led the study design and prepared the manuscript. XZ, ZW, and QW performed the research. XZ wrote the manuscript. SZ revised the manuscript. All authors contributed to the article and approved the submitted version.

## References

[1] Beird HC Bielack SS Flanagan AM Gill J Heymann D Janeway KA. Osteosarcoma. Nat Rev Dis Primers (2022) 8:77. doi: 10.1038/s41572-022-00409-y 36481668

[2] Gill J Gorlick R. Advancing therapy for osteosarcoma. Nat Rev Clin Oncol (2021) 18:609–24. doi: 10.1038/s41571-021-00519-8 34131316

[3] Siegel RL Miller KD Jemal A. Cancer statistics, 2019. CA Cancer J Clin (2019) 69:7–34. doi: 10.3322/caac.21551 30620402

[4] Lagmay JP Krailo MD Dang H Kim A Hawkins DS Beaty O 3rd. Outcome of patients with recurrent osteosarcoma enrolled in seven phase II trials through children’s cancer group, pediatric oncology group, and children’s oncology group: learning from the past to move forward. J Clin Oncol (2016) 34:3031–8. doi: 10.1200/JCO.2015.65.5381 PMC5012712 27400942

[5] Strauss SJ Ng T Mendoza-Naranjo A Whelan J Sorensen PH. Understanding micrometastatic disease and Anoikis resistance in ewing family of tumors and osteosarcoma. Oncologist (2010) 15:627–35. doi: 10.1634/theoncologist.2010-0093 PMC3227993 20479280

[6] Chen C Xie L Ren T Huang Y Xu J Guo W. Immunotherapy for osteosarcoma: Fundamental mechanism, rationale, and recent breakthroughs. Cancer Lett (2021) 500:1–10. doi: 10.1016/j.canlet.2020.12.024 33359211

[7] Adeshakin FO Adeshakin AO Afolabi LO Yan D Zhang G Wan X. Mechanisms for modulating anoikis resistance in cancer and the relevance of metabolic reprogramming. Front Oncol (2021) 11:626577. doi: 10.3389/fonc.2021.626577 33854965 PMC8039382

[8] Khan SU Fatima K Malik F. Understanding the cell survival mechanism of anoikis-resistant cancer cells during different steps of metastasis. Clin Exp Metastasis (2022) 39:715–26. doi: 10.1007/s10585-022-10172-9 35829806

[9] Sattari Fard F Jalilzadeh N Mehdizadeh A Sajjadian F Velaei K. Understanding and targeting anoikis in metastasis for cancer therapies. Cell Biol Int (2023) 47:683–98. doi: 10.1002/cbin.11970 36453448

[10] Jin L Chun J Pan C Kumar A Zhang G Ha Y. The PLAG1-GDH1 axis promotes anoikis resistance and tumor metastasis through camKK2-AMPK signaling in LKB1-deficient lung cancer. Mol Cell (2018) 69:87–99.e7. doi: 10.1016/j.molcel.2017.11.025 29249655 PMC5777230

[11] Corbet C Bastien E Santiago de Jesus JP Dierge E Martherus R Vander Linden C. TGFβ2-induced formation of lipid droplets supports acidosis-driven EMT and the metastatic spreading of cancer cells. Nat Commun (2020) 11:454. doi: 10.1038/s41467-019-14262-3 31974393 PMC6978517

[12] Zheng Q Yang Q Zhou J Gu X Zhou H Dong X. Immune signature-based hepatocellular carcinoma subtypes may provide novel insights into therapy and prognosis predictions. Cancer Cell Int (2021) 21:330. doi: 10.1186/s12935-021-02033-4 34193146 PMC8243542

[13] Yu S Hu C Cai L Du X Lin F Yu Q. Seven-gene signature based on glycolysis is closely related to the prognosis and tumor immune infiltration of patients with gastric cancer. Front Oncol (2020) 10:1778. doi: 10.3389/fonc.2020.01778 33072557 PMC7531434

[14] Chen W Gao C Liu Y Wen Y Hong X Huang Z. Bioinformatics analysis of prognostic miRNA signature and potential critical genes in colon cancer. Front Genet (2020) 11:478. doi: 10.3389/fgene.2020.00478 32582275 PMC7296168

[15] Lin Z Xu Q Miao D Yu F. An inflammatory response-related gene signature can impact the immune status and predict the prognosis of hepatocellular carcinoma. Front Oncol (2021) 11:644416. doi: 10.3389/fonc.2021.644416 33828988 PMC8019928

[16] Li H Li M Tang C Xu L. Screening and prognostic value of potential biomarkers for ovarian cancer. Ann Transl Med (2021) 9:1007. doi: 10.21037/atm-21-2627 34277807 PMC8267297

[17] Tang Y Guo C Yang Z Wang Y Zhang Y Wang D. Identification of a tumor immunological phenotype-related gene signature for predicting prognosis, immunotherapy efficacy, and drug candidates in hepatocellular carcinoma. Front Immunol (2022) 13:862527. doi: 10.3389/fimmu.2022.862527 35493471 PMC9039265

[18] Lv W Tan Y Zhou X Zhang Q Zhang J Wu Y. Landscape of prognosis and immunotherapy responsiveness under tumor glycosylation-related lncRNA patterns in breast cancer. Front Immunol (2022) 13:989928. doi: 10.3389/fimmu.2022.989928 36189319 PMC9520571

[19] Song W Ren J Xiang R Kong C Fu T. Identification of pyroptosis-related subtypes, the development of a prognosis model, and characterization of tumor microenvironment infiltration in colorectal cancer. Oncoimmunology (2021) 10:1987636. doi: 10.1080/2162402X.2021.1987636 34676149 PMC8526024

[20] Bielack SS Kempf-Bielack B Delling G Exner GU Flege S Helmke K. Prognostic factors in high-grade osteosarcoma of the extremities or trunk: an analysis of 1,702 patients treated on neoadjuvant cooperative osteosarcoma study group protocols. J Clin Oncol (2002) 20:776–90. doi: 10.1200/JCO.2002.20.3.776 11821461

[21] Zhang T Wang B Su F Gu B Xiang L Gao L. TCF7L2 promotes anoikis resistance and metastasis of gastric cancer by transcriptionally activating PLAUR. Int J Biol Sci (2022) 18:4560–77. doi: 10.7150/ijbs.69933 PMC9295057 35864968

[22] Shonibare Z Monavarian M O’Connell K Altomare D Shelton A Mehta S. Reciprocal SOX2 regulation by SMAD1-SMAD3 is critical for anoikis resistance and metastasis in cancer. Cell Rep (2022) 40:111066. doi: 10.1016/j.celrep.2022.111066 35905726 PMC9899501

[23] Gray-Owen SD Blumberg RS. CEACAM1: contact-dependent control of immunity. Nat Rev Immunol (2006) 6:433–46. doi: 10.1038/nri1864 16724098

[24] Nagaishi T Pao L Lin SH Iijima H Kaser A Qiao SW. SHP1 phosphatase-dependent T cell inhibition by CEACAM1 adhesion molecule isoforms. Immunity (2006) 25:769–81. doi: 10.1016/j.immuni.2006.08.026 17081782

[25] Pinkert J Boehm HH Trautwein M Doecke WD Wessel F Ge Y. T cell-mediated elimination of cancer cells by blocking CEACAM6-CEACAM1 interaction. Oncoimmunology (2021) 11:2008110. doi: 10.1080/2162402X.2021.2008110 35141051 PMC8820806

[26] To SKY Tang MKS Tong Y Zhang J Chan KKL Ip PPC. A selective β-catenin-metadherin/CEACAM1-CCL3 axis mediates metastatic heterogeneity upon tumor-macrophage interaction. Adv Sci (Weinh) (2022) 9:e2103230. doi: 10.1002/advs.202103230 35403834 PMC9165500

[27] Shapira R Weber JS Geva R Sznol M Kluger HM Wong DJ. A phase I, open-label, multicenter, single-dose escalation and multi-dose study of a monoclonal antibody targeting CEACAM1 in subjects with selected advanced or recurrent malignancies. J Clin Oncol (2020) 38:3094. doi: 10.1200/JCO.2020.38.15_suppl.3094

[28] Li B Wang Z Wu H Xue M Lin P Wang S. Epigenetic regulation of CXCL12 plays a critical role in mediating tumor progression and the immune response in osteosarcoma. Cancer Res (2018) 78:3938–53. doi: 10.1158/0008-5472.CAN-17-3801 29735547

[29] Wang Z Moresco P Yan R Li J Gao Y Biasci D. Carcinomas assemble a filamentous CXCL12-keratin-19 coating that suppresses T cell-mediated immune attack. Proc Natl Acad Sci U.S.A. (2022) 119:e2119463119. doi: 10.1073/pnas.2119463119 35046049 PMC8794816

[30] Mezzapelle R Leo M Caprioglio F Colley LS Lamarca A Sabatino L. CXCR4/CXCL12 activities in the tumor microenvironment and implications for tumor immunotherapy. Cancers (Basel) (2022) 14:2314. doi: 10.3390/cancers14092314 35565443 PMC9105267

[31] Liu X Wang Z Liu M Zhi F Wang P Liu X. Identification of LTF as a prognostic biomarker for osteosarcoma. J Oncol (2022) 2022:4656661. doi: 10.1155/2022/4656661 35096061 PMC8799371

[32] Zhu Y Chen B Yan J Zhao W Dou P Sun N. BNIP3 upregulation characterizes cancer cell subpopulation with increased fitness and proliferation. Front Oncol (2022) 12:923890. doi: 10.3389/fonc.2022.923890 35912211 PMC9326071

[33] Vianello C Cocetta V Catanzaro D Dorn GW 2nd De Milito A Rizzolio F. Cisplatin resistance can be curtailed by blunting Bnip3-mediated mitochondrial autophagy. Cell Death Dis (2022) 13:398. doi: 10.1038/s41419-022-04741-9 35459212 PMC9033831

[34] Khadiullina R Mirgayazova R Davletshin D Khusainova E Chasov V Bulatov E. Assessment of Thermal Stability of Mutant p53 Proteins via Differential Scanning Fluorimetry. Life (2022) 13:31. doi: 10.3390/life13010031 36675980 PMC9862671

[35] Ichimura K Bolin MB Goike HM Schmidt EE Moshref A Collins VP. Deregulation of the p14ARF/MDM2/p53 pathway is a prerequisite for human astrocytic gliomas with G1-S transition control gene abnormalities. Cancer Res (2000) 60:417–24. 10667596

[36] Xie L Yang Y Guo W Che D Xu J Sun X. The clinical implications of tumor mutational burden in osteosarcoma. Front Oncol (2021) 10:595527. doi: 10.3389/fonc.2020.595527 33898301 PMC8059407

[37] Zhang H Mao JS Hu WF. Functional genetic single-nucleotide polymorphisms (SNPs) in cyclin-dependent kinase inhibitor 2A/B (CDKN2A/B) locus are associated with risk and prognosis of osteosarcoma in chinese populations. Med Sci Monit (2019) 25:1307–13. doi: 10.12659/MSM.915001 PMC6391859 30774116

[38] Kawase T Ohki R Shibata T Tsutsumi S Kamimura N Inazawa J. PH domain-only protein PHLDA3 is a p53-regulated repressor of Akt. Cell (2009) 136:535–50. doi: 10.1016/j.cell.2008.12.002 19203586

[39] Wang X Li G Koul S Ohki R Maurer M Borczuk A. PHLDA2 is a key oncogene-induced negative feedback inhibitor of EGFR/ErbB2 signaling via interference with AKT signaling. Oncotarget (2015) 9:24914–26. doi: 10.18632/oncotarget.3674 PMC5982771 29861842

[40] Yao J Reyimu A Sun A Duoji Z Zhou W Liang S. UCHL1 acts as a potential oncogene and affects sensitivity of common anti-tumor drugs in lung adenocarcinoma. World J Surg Oncol (2022) 20:153. doi: 10.1186/s12957-022-02620-3 35546675 PMC9092673

[41] Mondal M Conole D Nautiyal J Tate EW. UCHL1 as a novel target in breast cancer: emerging insights from cell and chemical biology. Br J Cancer (2022) 126:24–33. doi: 10.1038/s41416-021-01516-5 34497382 PMC8727673

[42] Subramaniam D Angulo P Ponnurangam S Dandawate P Ramamoorthy P Srinivasan P. Suppressing STAT5 signaling affects osteosarcoma growth and stemness. Cell Death Dis (2020) 11:149. doi: 10.1038/s41419-020-2335-1 32094348 PMC7039889

[43] Li GZ Deng JF Qi YZ Liu R Liu ZX. COLEC12 regulates apoptosis of osteosarcoma through Toll-like receptor 4-activated inflammation. J Clin Lab Anal (2020) 34:e23469. doi: 10.1002/jcla.23469 32822099 PMC7676208

[44] Wang C Jing J Hu X Yu S Yao F Li Z. Gankyrin activates the hedgehog signalling to drive metastasis in osteosarcoma. J Cell Mol Med (2021) 25:6232–41. doi: 10.1111/jcmm.16576 PMC8366451 34089292

[45] Pan X Tan J Tao T Zhang X Weng Y Weng X. LINC01123 enhances osteosarcoma cell growth by activating the Hedgehog pathway via the miR-516b-5p/Gli1 axis. Cancer Sci (2021) 112:2260–71. doi: 10.1111/cas.14913 PMC8177773 33837611

[46] Qian H Lei T Hu Y Lei P. Expression of lipid-metabolism genes is correlated with immune microenvironment and predicts prognosis in osteosarcoma. Front Cell Dev Biol (2021) 9:673827. doi: 10.3389/fcell.2021.673827 33937273 PMC8085431

[47] Li Y Wang JS Zhang T Wang HC Li LP. Identification of new therapeutic targets for gastric cancer with bioinformatics. Front Genet (2020) 11:865. doi: 10.3389/fgene.2020.00865 33014013 PMC7461879

[48] Zhong Y Zhang Y Wei S Chen J Zhong C Cai W. Dissecting the effect of sphingolipid metabolism gene in progression and microenvironment of osteosarcoma to develop a prognostic signature. Front Endocrinol (Lausanne) (2022) 13:1030655. doi: 10.3389/fendo.2022.1030655 36313783 PMC9613955

[49] Yook YH Kang KH Maeng O Kim TR Lee JO Kang KI. Nitric oxide induces BNIP3 expression that causes cell death in macrophages. Biochem Biophys Res Commun (2004) 321:298–305. doi: 10.1016/j.bbrc.2004.06.144 15358175

[50] Lamy L Ticchioni M Rouquette-Jazdanian AK Samson M Deckert M Greenberg AH. CD47 and the 19 kDa interacting protein-3 (BNIP3) in T cell apoptosis. J Biol Chem (2003) 278:23915–21. doi: 10.1074/jbc.M301869200 12690108

[51] Bernardini G Sciumè G Bosisio D Morrone S Sozzani S Santoni A. CCL3 and CXCL12 regulate trafficking of mouse bone marrow NK cell subsets. Blood (2008) 111:3626–34. doi: 10.1182/blood-2007-08-106203 18227348

[52] Sánchez-Martín L Estecha A Samaniego R Sánchez-Ramón S Vega MÁ Sánchez-Mateos P. The chemokine CXCL12 regulates monocyte-macrophage differentiation and RUNX3 expression. Blood (2011) 117:88–97. doi: 10.1182/blood-2009-12-258186 20930067

[53] Tiberti S Catozzi C Croci O Ballerini M Cagnina D Soriani C. GZMKhigh CD8+ T effector memory cells are associated with CD15high neutrophil abundance in non-metastatic colorectal tumors and predict poor clinical outcome. Nat Commun (2022) 13(1):6752. doi: 10.1038/s41467-022-34467-3 36347862 PMC9643357

[54] Ahn S Choi HB Kim TG. HLA and disease associations in Koreans. Immune Netw (2011) 11:324–35. doi: 10.4110/in.2011.11.6.324 PMC3275700 22346771

[55] Luo Z Zhang H Xiao Y Wang R Zhang L Huang C. Durable response to immunotherapy with antiangiogenic drug in large-cell lung carcinoma with multiple fulminant postoperative metastases: A case report. Front Oncol (2021) 11:633446. doi: 10.3389/fonc.2021.633446 34094914 PMC8173040

[56] Deichaite I Hopper A Krockenberger L Sears TJ Sutton L Ray X. Germline genetic biomarkers to stratify patients for personalized radiation treatment. J Transl Med (2022) 20:360. doi: 10.1186/s12967-022-03561-x 35962345 PMC9373374

[57] Luo Y Deng Z Chen J. Pivotal regulatory network and genes in osteosarcoma. Arch Med Sci (2013) 9:569–75. doi: 10.5114/aoms.2012.30956 PMC3701964 23847684

[58] Endo-Munoz L Cumming A Sommerville S Dickinson I Saunders NA. Osteosarcoma is characterised by reduced expression of markers of osteoclastogenesis and antigen presentation compared with normal bone. Br J Cancer (2010) 103:73–81. doi: 10.1038/sj.bjc.6605723 20551950 PMC2905286

[59] Fu Q Agarwal D Deng K Matheson R Yang H Wei L. An unbiased machine learning exploration reveals gene sets predictive of allograft tolerance after kidney transplantation. Front Immunol (2021) 12:695806. doi: 10.3389/fimmu.2021.695806 34305931 PMC8297499

[60] Verma NK Wong BHS Poh ZS Udayakumar A Verma R Goh RKJ. Obstacles for T-lymphocytes in the tumour microenvironment: Therapeutic challenges, advances and opportunities beyond immune checkpoint. EBioMedicine (2022) 83:104216. doi: 10.1016/j.ebiom.2022.104216 35986950 PMC9403334

[61] Zhang Y Wang Y Ying L Tao S Shi M Lin P. Regulatory role of N6-methyladenosine (m6A) modification in osteosarcoma. Front Oncol (2021) 11:683768. doi: 10.3389/fonc.2021.683768 34094986 PMC8170137

[62] Tawbi HA Burgess M Bolejack V Van Tine BA Schuetze SM Hu J. Pembrolizumab in advanced soft-tissue sarcoma and bone sarcoma (SARC028): A multicentre, two-cohort, single-arm, open-label, phase 2 trial. Lancet Oncol (2017) 18:1493–501. doi: 10.1016/S1470-2045(17)30624-1 PMC7939029 28988646

[63] Takenaka W Takahashi Y Tamari K Minami K Katsuki S Seo Y. Radiation dose escalation is crucial in anti-CTLA-4 antibody therapy to enhance local and distant antitumor effect in murine osteosarcoma. Cancers (Basel) (2020) 12:1546. doi: 10.3390/cancers12061546 32545427 PMC7352693

[64] Lussier DM Johnson JL Hingorani P Blattman JN. Combination immunotherapy with α-CTLA-4 and α-PD-L1 antibody blockade prevents immune escape and leads to complete control of metastatic osteosarcoma. J Immunother Cancer (2015) 3:21. doi: 10.1186/s40425-015-0067-z 25992292 PMC4437699

[65] Tian H Cao J Li B Nice EC Mao H Zhang Y. Managing the immune microenvironment of osteosarcoma: the outlook for osteosarcoma treatment. Bone Res (2023) 11:11. doi: 10.1038/s41413-023-00246-z 36849442 PMC9971189

